# Pancreatic Resections for Advanced M1-Pancreatic Carcinoma: The Value of Synchronous Metastasectomy

**DOI:** 10.1155/2010/579672

**Published:** 2010-12-14

**Authors:** S. K. Seelig, B. Burkert, A. M. Chromik, A. Tannapfel, W. Uhl, M. H. Seelig

**Affiliations:** ^1^Department of Gynecology, North-West-Hospital Frankfurt, Academic Teaching Hospital, University of Frankfurt, Steinbacher Hohl 2-26, 60488 Frankfurt, Germany; ^2^Department of General Surgery, St. Joseph Hospital, Ruhr-University, Gudrunstra*β*e 56, 44791 Bochum, Germany; ^3^Institute for Pathology, BG Kliniken Bergmannsheil, Ruhr-University, Buerkle-de-la-Camp-Platz 1, 44789 Bochum, Germany; ^4^Department of Surgery, Clinics of the Main Taunus County, Academic Teaching Hospital, University of Frankfurt, Kronberger Stra*β*e 36, 65812 Bad Soden, Germany

## Abstract

*Background*. For M1 pancreatic adenocarcinomas pancreatic resection is usually not indicated. However, in highly selected patients synchronous metastasectomy may be appropriate together with pancreatic resection when operative morbidity is low. *Materials and Methods*. From January 1, 2004 to December, 2007 a total of 20 patients with pancreatic malignancies were retrospectively evaluated who underwent pancreatic surgery with synchronous resection of hepatic, adjacent organ, or peritoneal metastases for proven UICC stage IV periampullary cancer of the pancreas. Perioperative as well as clinicopathological parameters were evaluated. *Results*. There were 20 patients (9 men, 11 women; mean age 58 years) identified. The primary tumor was located in the pancreatic head (*n* = 9, 45%), in pancreatic tail (*n* = 9, 45%), and in the papilla Vateri (*n* = 2, 10%). Metastases were located in the liver (*n* = 14, 70%), peritoneum (*n* = 5, 25%), and omentum majus (*n* = 2, 10%). Lymphnode metastases were present in 16 patients (80%). All patients received resection of their tumors together with metastasectomy. Pylorus preserving duodenopancreatectomy was performed in 8 patients, distal pancreatectomy in 8, duodenopancreatectomy in 2, and total pancreatectomy in 2. Morbidity was 45% and there was no perioperative mortality. Median postoperative survival was 10.7 months (2.6–37.7 months) which was not significantly different from a matched-pair group of patients who underwent pancreatic resection for UICC adenocarcinoma of the pancreas (median survival 15.6 months; *P* = .1). *Conclusion*. Pancreatic resection for M1 periampullary cancer of the pancreas can be performed safely in well-selected patients. However, indication for surgery has to be made on an individual basis.

## 1. Introduction

Surgery for pancreatic cancer is probably the most demanding and risky operative procedure in abdominal surgery [1]. Remarkable progress has been achieved during the last three decades and pancreatic surgery can nowadays be performed safely with low morbidity and mortality in various specialized hospitals around the world. This is in part a result of regionalization of pancreatic surgery into high volume centers [2] resulting in an increased resection rate in the presence of pancreatic carcinoma which is nowadays approximately 60% and to an extension of indications for resection. Consequently, vascular resection, considered a contraindication for pancreatic resection a couple of years ago, is now implemented in the armamentarium of pancreatic surgeons to realize R0-resections when clearance of the portal or superior mesenteric vein can not be realized without vascular resection. 

The results following synchronous resection of liver metastasis together with resection of periampullary or pancreatic adenocarcinoma or pancreatic resection for M1 carcinoma are not equivocal. Although these procedures can be performed safely, the median survival time ranges between 6 and 13 months [3, 4]. Therefore, synchronous metastasectomy of periampullary cancer is rarely performed when extended disease has already been found preoperatively.

However, within daily clinical practice different scenarios occur which may require a decision for resection in an M1 situation. In a young patient with advanced disease, resection may provide a small but important increase of survival. In addition, metastatic disease may become overt when the point of no return has already been passed as it may be the case in the presence of positive interaortocaval lymphnodes, or metastatic disease will be detected during operation despite negative imaging results preoperatively. Finally, a strong patient desire may force the surgeon to go further than consented guidelines may summarize existing evidence. Since curative R0 resection is the most important prognostic factor for long-term survival even a small potential to achieve R0 resection may provide the legitimisation for extended resection despite the fact that overt systemic disease is already present. 

So far, there is little data available on the value of synchronous metastasectomy together with pancreatic resection in patients with pancreatic carcinoma. Patients who undergo resection of metastases together with pancreatic resection seem to have a higher complication rate than patients with multivisceral liver resections [5]. An extended lymphadenectomy is associated with an increased postoperative complication rate, but has no impact on long-term survival [6].

The fact that despite R0 resection long-term survival does not exceed 25% even in the most experienced pancreatic centers may prove that carcinoma of the pancreas is a systemic disease. Further improvement of survival can only be achieved by adjuvant treatment [7] (Picozzi et al., 2003). It seems logical, that in the presence of metastatic disease a low tumor load may increase the success of adjuvant chemotherapy although the absolute increase in survival time may be very low. We therefore hypothesized that radical resection of M1-pancreatic carcinoma may be justified in well-selected patients as long as these operations can be performed with a low morbidity and mortality.

Thus, we aimed at reviewing our experiences with pancreatic resections in patients with M1 periampullary cancer of the pancreas after having launched a new pancreatic program in our hospital.

## 2. Patients and Methods

Between January 2004 and December 2007, 470 patients underwent surgery for pancreatic cancer. There were 280 patients who underwent either pancreatoduodenectomy or left-sided pancreatic resection for histologically proven pancreatic adenocarcinoma or periampullary cancer. Among this patient population we identified 20 (7,1%) patients (9 men, 11 women; mean age 60 years) in our prospective database who underwent either a pancreaticoduodenectomy (PD), or a left-sided distal pancreatectomy together with resection of metastasis for pancreatic adenocarcinoma. Resection of metastatic disease was defined as the resection of any tumor deposit outside the regional lymph nodes or resection of infiltrated surrounding organs in order to achieve a R0 resection. Patients with neuroendocrine tumors or metastatic disease into the pancreas were not considered.

Perioperative imaging included a computed tomography scanning and endoscopic ultrasound in all instances. Magnetic resonance imaging, magnetic resonance cholangiography, or endoscopic retrograde cholangiopancreatography were performed when indicated.

In patients who were found intraoperatively to harbour metastatic disease the decision for resection was based on the impression to reach a R0 situation with synchronous resection of metastasis and a good clinical performance status of ASA III or better. Patients with suspected metastatic disease preoperatively were operated with potentially curative intention or with palliative intention. Reasons for resection were good performance status and patient's will to receive maximal treatment.

All pathologic specimen were reviewed by a single pathologist (AT) to confirm the diagnosis of pancreatic adenocarcinoma or cancer of the papilla Vateri. 

The overall incidence of postoperative complications was evaluated. A pancreatic fistula was defined as a prolonged drainage with amylase activity of more than 10.000 U/L. Wound infection was defined as any wound requiring reopening for the drainage of pus together with a positive wound culture. Mortality was defined as any death during postoperative hospitalization or within 30 days of surgery. Follow-up information was obtained through direct contact with the patient and review of hospital charts and operative notes. Follow-up data was complete for every patient (100%). 

The results of the study group were compared with a matched-pair control group of 20 patients with pancreatic adenocarcinoma, who were matched according to age and tumor location and who did undergo only pancreaticoduodenectomy or distal pancreatic resection for stadium IIb or III pancreatic adenocarcinoma.

### 2.1. Statistical Analysis

All statistical analyses were performed using SPSS-software (version 15). The distribution of age at operation, blood loss, and postoperative hospital stay were presented as median with range. Comparisons between subgroups were performed with Fisher's exact test and differences between means were tested by the *t*-test. Cases were matched with control group (resection without multivisceral resection) in a 1 : 1 fashion. Specifically, cases and controls were matched on primary tumor characteristics and age of the patient. Morbidity, mortality, and overall survival were compared between cases and control group. The nonparametric product limit method (Kaplan-Meier estimations) was used to analyse overall survival from the date of surgery. Differences in survival were examined using the log-rank test. Two-sided *P* values were always computed and an effect was considered statistically significant at *P* ≤ .05.

### 2.2. Results

#### 2.2.1. Patient Data

The primary tumor was located in the pancreatic head in 9 (45%) patients, in the pancreatic tail in 9 (45%), and at the papilla Vateri in 2 (10%). Synchronous liver metastasis was present in 14 (70%) patients, 4 (20%) patients had peritoneal metastases, one patient had a metastasis in the transverse mesocolon, 2 (10%) patients had a metastasis in the greater omentum, and 3 (15%) patients had macroscopically lymph node metastases. Six patients (30%) had metastatic disease at more than one location. In 7 (35%) patients the tumor was locally advanced with infiltration of stomach or pylorus. Metastatic disease was known before operation based on preoperative imaging in 5 of 20 patients (25%). 

Details of the patients are given in Table 1.

#### 2.2.2. Operations

Of the 20 patients, 8 (40%) underwent a pylorus-preserving duodenopancreatectomy and 2 (10%) a classic Whipple-procedure. In 8 (40%) patients a distal pancreatic resection was performed and 2 (10%) patients underwent a total pancreatectomy. 

Details of the localisation of the tumor and the metastases, TNM-staging, R-status postoperatively, and type of surgery performed are listed in Table 2.

An R0/R1-resection could be achieved in 11 patients (55%), whereas in the remaining patients only an R2 resection was performed due to remaining metastatic disease in liver.

#### 2.2.3. Perioperative Data

There was no perioperative mortality, and complications occurred in 9 (45%) patients. The median intraoperative blood loss was 1000 mL (range 300–2500 mL) and the median postoperative length of hospital stay was 20.7 d (11–71 d). This was statistically not different to a matched-pair group of patients with ductal adenocarcinoma of the pancreas without metastatic disease (Tables 3 and 4). All patients received postoperative adjuvant or palliative chemotherapy, two patients underwent neoadjuvant radiochemotherapy prior to surgery.

#### 2.2.4. Survival

**Figure 1:**
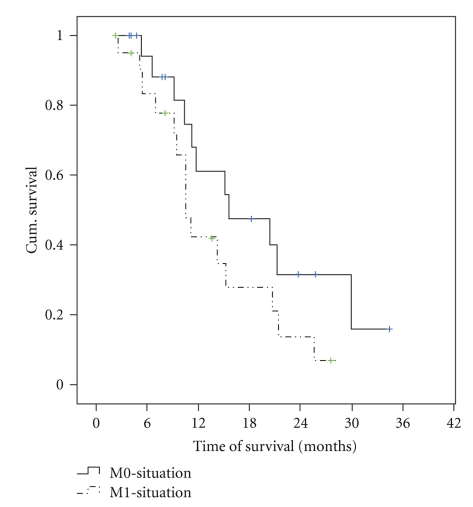
Kaplan-Meier curve comparing survival curve of patients with metastases and patients who did not have metastases (*P* = .11; log-rank test).

The median postoperative survival was 10.7 months (range 2.6–37.8 mo) which was not significantly different to the control group who had a median survival time of 15.6 months (*P* = .11; Figure 1). All deaths were caused by recurrent cancer.There was no difference in the median survival between patients with liver metastases (median survival time 11 months) compared to patients who had metastases at other locations (median survival 14.1 months; *P* = .62).

## 3. Discussion

Pancreatic cancer still carries a dismal prognosis and the potential to cure a patient can only be achieved when the primary tumor can be completely resected. However, this scenario can only be accomplished in about 5%–25% of patients presenting with locally resectable cancer. Patients with metastatic pancreatic cancer who receive palliative chemotherapy with gemcitabine have a median survival time of 5.6 months [8]. Various protocols with gemcitabine-based regimen or multidrug regimen tested in prospectively randomised phase III clinical trials have not altered this situation substantially making metastatic pancreatic cancer one of the most frustrating malignancies to investigate and treat [9]. Therefore, when metastatic disease is recognized preoperatively, an operative procedure is usually avoided except for surgical palliation. In addition, according to the S-3 guidelines of the German Cancer Society pancreatic resections should be avoided in the presence of intraoperative metastatic disease [10]. However, locally advanced disease may be resected as long as an R0 resection can be achieved. Both statements have a grade of evidence of 3 which means that only systematic reviews and individual case control studies are available to scientifically support this statement. It seems reasonable, that postoperative chemotherapy may be more effective, when no gross tumor is left in situ. In addition, the high recurrence rate even after R0 resection is an indirect prove for early systemic spread which is not treatable by surgery but by chemotherapy.

This demonstrates that additional data is required to solve this issue. Our study gives support to the hypothesis—despite the relatively small number of patients—that in the presence of metastasis of a pancreatic adenocarcinoma radical resection is possible and safe and that overall survival is comparable to patients who do not have metastatic disease. This relatively good result is in part consequence of restriction of the procedure to well suited patients who were expected to tolerate even a significantly expanded procedure. The mean age of our patients was 58 years, and all patients were either ASA 1 or 2 (data not given). Therefore, it was expected that these patients would tolerate pancreatic resection with synchronous metastasectomy without significant increase in morbidity or mortality. In addition, every patient received chemotherapy postoperatively. Nevertheless, the median survival of 10.6 months is low, but we believe that we could offer these patients additional life time, although quality of life was not evaluated. However, these procedures should be restricted to well-suited patients and only be performed at centers with significant expertise in pancreatic surgery.

 A shortcoming of the study may be the fact that patients had various presentation of the M1 category. Most of our patients had liver metastasis, but there were also patients with peritoneal or lymph nodes metastases who were classified as M1 or the tumor involved surrounding organs. Since there is no significant difference in survival according to the location of the metastasis of pancreatic carcinoma, we believe that an analysis of this group was appropriate.

According to the results of at least 3 randomized trials extended lymphadenectomy has not been effective in improving survival of patients with pancreatic adenocarcinoma with 1 and 3 years survival between 51% and 77% and between 16 and 41%, respectively [6]. Therefore, in an nonmetastasized pancreatic adenocarcinoma a standard lymphadenectomy is adequate. 

When locally advanced cancer with suspected venous infiltration is present extensive surgery can be performed safely with morbidity and mortality rates comparable to conventional resections. In a recent analysis of 136 patients with locally advanced pancreatic cancer Yekebas et al. could demonstrate that patients with concomitant vascular resections had the same median (15 versus 16 mo; *P* = .86) and two year survival (34% versus 36%: *P* = .9) compared to patients who did not undergo vascular resection [11]. Even in multivariate analysis vascular infiltration was not considered a negative prognostic factor. Even arterial encasement of the celiac trunk in the presence of a pancreatic corpus carcinoma is not considered a contraindication for surgery. Hirano et al. recently published a series of 23 patients with pancreatic body cancer with infiltration of the celiac trunk. Negative surgical margins were obtained in 91% of patients, postoperative mortality was 0%, and the median survival was 21 months [12]. However, early hepatic recurrence in 6 patients was a hint that this procedure may be indicated for the treatment of less advanced disease.

 In a recently published study Shrikhande et al. reported a series of 29 patients with M1 ductal adenocarcinoma of the pancreas who underwent pancreatic resection with synchronous resection of metastases. Of these 11 patients had metastatic disease to the liver and 9 to the peritoneum. The median survival following resection was 11.4 months and 12.9 months, respectively [4]. The authors concluded, that concomitant resection of primary pancreatic tumor and metastases can be performed safely. However, a risk benefit ratio should be carefully assessed since the overall increase in survival is moderate. In our study the survival time of patients with liver metastasis was 11 months compared to 14.1 months when metastases were located at other regions (*P* = .62). These values are comparable to the values published by Shrikhande et al. [4]. 

In a study of 22 patients who underwent pancreatic resection and synchronous liver resection for metastasised pancreatic adenocarcinoma, Gleisner et al. found that the median survival was only 5.9 months, which was not statistically different from patients who underwent palliative bypass alone (5.6 months; *P* = .46) [3]. They concluded from the results of their study that simultaneous resection for patients who present with synchronous liver metastasis from pancreatic adenocarcinoma is not justified. In our study we found a median survival of 10.6 months for the complete group, while the 14 patients with liver metastases had a median survival of 11 months. Despite this small increase we believe that in highly selected patients after careful counselling this aggressive therapy is still an option that should be offered to the patient.

The fact that there was no significant difference in survival between the study and the matched-pair control group despite an objective difference in the median survival of 5 months is probably due to the limited number of patients. With an increased number of patients we would have expect this difference to reach statistical significance. Compared to published results of patients receiving palliative chemotherapy who have a median survival time of approximately 5.4–8.4 months [13, 14], the increase in survival in patients with M1 ductal adenocarcinoma of the pancreas is about 2-5 months. This increase is not substantial to recommend this procedure in general. We believe that patients who may benefit must be carefully selected and therapeutic options and expected survival should be discussed with the patients and their families. In addition, the perioperative morbidity and mortality must be low.

In the presence of pancreatic neuroendocrine carcinomas extended resections or even debulking operations seem to be associated with increased survival. Sarmiento et al. reported on a series of 23 patients with pancreatic islet-cell carcinomas with synchronous hepatic metastases who underwent synchronous hepatic resections without perioperative mortality. 9 patients were resected R0/R1 and 14 had a R2-resection. The overall survival was 71% with a median survival time of 76 months [15]. This demonstrates, that extended resections in this subset of metastasized patients with primary pancreatic tumors are justified. The preoperative or intraoperative differentiation between a pancreatic adenocarcinoma and a neuroendocrine carcinoma may be difficult when typical carcinoma associated syndromes are missing preoperatively. However, intraoperatively the differentiation may be easier from the macroscopic point of view, and when histology has not been determined preoperatively, intraoperative frozen section may identify the tumor so operative strategy can be planned appropriately. 

Currently, a monocentric prospective randomized trial on the value of pancreatic resection in the presence of liver metastasis is under way in Germany (NCT00855634, PAMEVITUM). The results of this study may give further evidence whether pancreatic resection in this scenario is justified.

The results of our study demonstrate, that resection of pancreatic adenocarcinoma with synchronous metastases can be performed safely. Justification for combined resection should be made on an individual basis for each patient only. There is probably a small increase in survival compared to patients who undergo palliative chemotherapy and there was no statistically significant difference in survival compared to patients who underwent resection for stadium IIb or III tumors. The decision to resect a patient in an M1 situation should be carefully assessed and may be an option in highly selected cases.

## Figures and Tables

**Table 1:** Clinicopathologic characteristics (M1-Patients).

Variable	No. of patients (%) (*n* = 20)
Patient characteristics	
Mean age ± SD	58.4 ± 11.8
Sex (% men)	9 (45)
Median tumor size (cm)	4.1 (1–11)
Lymph node disease	16 (80)
Origin of tumor	
Pancreas head	9 (45)
Pancreas corpus/tail	9 (45)
Papilla	2 (10)
Hepatic metastasis	14 (70)
Preoperative known	9 (45)
unknown	5 (25)
Median size of largest metastasis (cm)	1,6 (0,5–4,0)
Solitary metastases	5 (25)
Other metastases	
Peritoneum	5 (25)
Omentum majus	2 (10)
Lymphnodes	4 (20)
Adjuvant chemotherapy	20 (100%)
(+neoadjuvant chemotherapy)	2 (10)
Median survival (months)	10.7 (2.6–37.8)

**Table 2:** Localisation of primary tumor, site of metastasis, TNM-staging, type of surgery, and survival of 20 patients with M1-pancreatic adenocarcinoma.

No.	Primary tumor	Site of metastasis	TNM	R	Type of surgery	Survival (mo)	Status
1	Pancreatic tail	Liver segment 5 and 6, peritoneum	T3N0M1	R2	Distal pancreatectomy, tumor debulking	5.3	Dead
2	Pancreatic head	Liver (multifocal), peritoneum, mesocolon transversum	T3N1M1	R2	pp-Whipple	21.3	Dead
3	Pancreatic body	Liver segment 2/3, peritoneum, stomach, mesocolon transversum, diaphragm	T3N1M1	R0	Distal pancreatic resection, gastrectomy, left hemicolectomy, liver segment 2/3	2.6	Dead
4	Pancreatic head	Liver segment 2/3, peritoneum	T3N1M1	R2	Whipple procedure, gastrectomy, right hemicolectomy	15.2	Dead
5	Pancreatic head	Liver segment 3	T3N1M1	R0	pp-Whipple, atyp. liver segment 3	9.3	Dead
6	Pancreatic tail	Peritoneum, ovary, infiltration of stomach, colon transversum and left kidney	T4N1M1	R2	Distal pancreatectomy, 2/3 gastric resection, left hemicolectomy, left nephrectomy, left adrenalectomy	5.1	Dead
7	Pancreatic body	Liver segment 3,5, infiltration of stomach and celiac trunk	T3N1M1	R0	Distal pancreatectomy, subtotal gastric resection, liver resection seg. 3 + 5	10.6	Dead
8	Pancreatic head	Omentum majus	T3N0M1	R0	Whipple-procedure, partial portal vein resection, omentectomy	20.6	Dead
9	Pancreatic head	Liver segment 4b	T3N1M1	R0	pp-Whipple, liver segment 4b	37.8	Alive
10	Pancreatic body	Liver segment 3, 4b, 5, peritoneum, portal vein and pylorus infiltration	T3N1M1	R1	Pancreatectomy, 2/3 gastric resection, atypical liver resection, partial portal vein resection	10.6	Dead
11	Pancreatic head	Liver segment 2, 3, 4	T3N1M1	R2	pp-Whipple, Biopsy segment 3	6.8	Dead
12	Pancreatic body	Liver bilobar	T3N1M1	R2	Distal pancreatic resection, liver segment 3 and 7	23.7	Alive
13	Pancreatic head	Liver segment 3	T3N1M1	R0	pp-Whipple, atypical liver segment 3	11	Dead
14	Papilla	Diffuse lymph node metastases	T3N1M1	R0	pp-Whipple, lymph node resection	18,2	Dead
15	Pancreatic tail	Stomach infiltration	T3N0M1	R0	Distal pancreatic resection, gastric resection	25.6	Dead
16	Pancreatic tail	Liver bilobar, Stomach infiltration	T3N0M1	R2	Distal pancreatic resection, gastric resection	14.3	Alive
17	Pancreatic head	Liver segment 4 a + b	T3N1M1	R0	pp-Whipple, atypical liver resection	12.5	
18	Pancreatic tail	Liver segment 5 + 8, metastasis stomach, infiltration of left adrenal and splenic artery	T3N1M1	R2	Distal pancreatic resection, left adrenalectomy, gastric resection	10.5	Dead
19	Pancreatic head	Infiltration of stomach and portal vein	T3N1M1	R0	Pancreatectomy, 2/3-gastric resection, portal vein resection	14.1	Dead
20	Papilla	Liver segment 3 + 4a	T4N1M1	R2	pp-Whipple, liver segment 3, 4a	8	Dead

(1) pp-Whipple: pylorus-preserving pancreaticoduodenectomy.

**Table 3:** Patient's clinicopathologic characteristics (M0-Patients).

Variable	No. of patient (%), *n* = 20
Patient characteristics	
Mean age ± SD	62.3 ± 12
Sex (% men)	11 (55)
Median tumor size (cm)	3.9 (1.3–7)
Lymph node disease	16 (80)
Origin of tumor	
Pancreas head	9 (45)
Pancreas corpus/tail	9 (45)
Papilla	2 (10)
Adjuvant chemotherapy	20 (100%)
Median survival (months)	15.6 (5.3–35.9)

**Table 4:** Operative details of study group (M1-Patients) and control group (M0).

	M1	M0	*P*-value
Intraoperative blood loss (mL)	1000 (300–2500)	1400 (500–5100)	.32
Main operative procedure			
Pylorus preserving Whipple	8 (40)	7	
Whipple	2 (10)	5	
Distal pancreatectomy	8 (40)	6	
Pancreatectomy	2 (2)	2	
Length of postoperative hospital stay (days)	20.7 (11–71)	25.1 (12–92)	.46
Complications	9 (45)	10 (50)	.76
